# Multiscale molecular profiling of pathological bone resolves sexually dimorphic control of extracellular matrix composition

**DOI:** 10.1242/dmm.048116

**Published:** 2021-03-17

**Authors:** Aikta Sharma, Alice Goring, Peter B. Johnson, Roger J. H. Emery, Eric Hesse, Alan Boyde, Bjorn R. Olsen, Andrew A. Pitsillides, Richard O. C. Oreffo, Sumeet Mahajan, Claire E. Clarkin

**Affiliations:** 1 School of Biological Sciences, Highfield Campus, University of Southampton, Southampton SO17 1BJ, UK; 2 School of Chemistry and Institute for Life Sciences, Highfield Campus, University of Southampton, Southampton SO17 1BJ, UK; 3 Department of Surgery and Cancer, Faculty of Medicine, St Mary's Campus, Imperial College London, London W2 1PG, UK; 4 Institute of Molecular Musculoskeletal Research, Faculty of Medicine, LMU Munich, Planegg-Martinsried, Munich 80336, Germany; 5 Dental Physical Sciences, Barts and The London School of Medicine and Dentistry, Queen Mary University of London, London E1 4NS, UK; 6 Department of Developmental Biology, Harvard School of Dental Medicine, Boston, MA 02115, USA; 7 Department of Comparative Biomedical Sciences, Royal Veterinary College, London NW1 0TU, UK; 8 Centre for Human Development, Stem Cell and Regeneration, Institute of Developmental Sciences, Faculty of Medicine, University of Southampton, Southampton SO16 6YD, UK

**Keywords:** Polarisation-resolved second-harmonic generation, Raman spectroscopy, Sexual dimorphism, Extracellular matrix, Collagen, VEGF

## Abstract

Collagen assembly during development is essential for successful matrix mineralisation, which determines bone quality and mechanocompetence. However, the biochemical and structural perturbations that drive pathological skeletal collagen configuration remain unclear. Deletion of vascular endothelial growth factor (VEGF; also known as VEGFA) in bone-forming osteoblasts (OBs) induces sex-specific alterations in extracellular matrix (ECM) conformation and mineralisation coupled to vascular changes, which are augmented in males. Whether this phenotypic dimorphism arises as a result of the divergent control of ECM composition and its subsequent arrangement is unknown and is the focus of this study. Herein, we used murine osteocalcin-specific *Vegf* knockout (OcnVEGFKO) and performed *ex vivo* multiscale analysis at the tibiofibular junction of both sexes. Label-free and non-destructive polarisation-resolved second-harmonic generation (p-SHG) microscopy revealed a reduction in collagen fibre number in males following the loss of VEGF, complemented by observable defects in matrix organisation by backscattered electron scanning electron microscopy. This was accompanied by localised divergence in collagen orientation, determined by p-SHG anisotropy measurements, as a result of OcnVEGFKO. Raman spectroscopy confirmed that the effect on collagen was linked to molecular dimorphic VEGF effects on collagen-specific proline and hydroxyproline, and collagen intra-strand stability, in addition to matrix carbonation and mineralisation. *Vegf* deletion in male and female murine OB cultures *in vitro* further highlighted divergence in genes regulating local ECM structure, including *Adamts2*, *Spp1*, *Mmp9* and *Lama1*. Our results demonstrate the utility of macromolecular imaging and spectroscopic modalities for the detection of collagen arrangement and ECM composition in pathological bone. Linking the sex-specific genetic regulators to matrix signatures could be important for treatment of dimorphic bone disorders that clinically manifest in pathological nano- and macro-level disorganisation.

This article has an associated First Person interview with the first author of the paper.

## Abstract (precis)

**Summary:** Combined application of polarisation-resolved second-harmonic generation microscopy and Raman spectroscopy identified unique, sexually dimorphic extracellular matrix signatures linking pathological matrix disorganisation with composition.

## INTRODUCTION

Bone formation prevalent during development and repair is controlled by the coordinated expression of genetic regulators of osteoblast (OB) behaviour and commitment, and is characterised by the formation of non-mineralised osteoid comprising predominantly type I collagen (reviewed in Blair et al., 2017). The three-dimensional configuration of the osteoid scaffold, organised in part by the OB-derived non-collagenous proteins, subsequently facilitates the nucleation of apatite crystals during the mineralisation process (Blair et al., 2017; Houston et al., 2004; Staines et al., 2012). Both collagen organisation and the extent of matrix mineralisation influence the mechanosensitivity of bone. The hydroxyapatite (HA) crystals, deposited during matrix mineralisation, can withstand up to four times the stress of the collagen fibrils, whereas the bulk collagen phase is responsible for providing flexibility and fracture resistance during the deformation response (Nair et al., 2013). This energy-dissipation property of type I collagen in bone is largely due to its structure, in which fibrils made up of bundles of triple helical tropocollagen molecules containing a glycine-X-Y motif are organised into macroscale fibres (Currey, 1988; Donnelly et al., 2010; Ramshaw et al., 1998; Shoulders and Raines, 2009). A number of studies have shown that direct alterations to collagen properties implicate the overall mechanical properties of bone and often result in changes to fracture risk (Diebold et al., 1991; Kafantari et al., 2000; Sroga and Vashishth, 2012; Suarez et al., 1996).

The preclinical administration of collagen peptides has been shown to increase both the organic component of bone (Watanabe-Kamiyama et al., 2010) and enhance the biomechanical resistance of vertebrae in rats (de Almeida Jackix et al., 2010). Furthermore, clinical studies have demonstrated that specific collagen peptides can increase the bone mineral density (BMD) in postmenopausal women subjected to age-related bone loss (König et al., 2018). Conversely, during bone pathophysiology, the failure of appropriate osteoid mineralisation translates to an abnormal accumulation and distribution of the collagen matrix. This effect is prevalent in skeletal conditions such as osteomalacia or ‘soft bones’, wherein the proportion of mineral to collagen is reduced (Berry et al., 2002), whereas in osteogenesis imperfecta (OI) or ‘brittle bone disease’, the deficient bone matrix is hypermineralised in patients carrying mutations in the genes encoding the type I procollagen chains (*COL1A1* or *COL1A2*) (Boyde et al., 1999; Forlino et al., 2011).

The skeletal system is profoundly sexually dimorphic, being on average larger in men than in women (reviewed in Seeman, 2001). Bone strength is determined by bone mass acquisition during the pubertal years and subsequent bone turnover, which underlie sex differences in bone length, BMD, shape and microarchitecture throughout life. Sex differences have been reported in diseases associated with mutations in collagen-encoding genes. In an OI mouse model, Yao et al. (2013) reported sexual dimorphism linked to the structural and mechanical properties of bone, suggesting that collagen-encoding genes exert a divergent influence upon male and female bones. Currently, little information exists on how collagen fibrils and mineral species interact at the molecular level, and how they influence the skeletal sexual dimorphism in bone development and disease. Identification of sex-specific mechanisms of collagen assembly could provide novel targets to therapeutically improve bone structure, strength and reduce fracture risk in skeletal pathology.

Structurally type I collagen fibrils in bone, ∼200 nm in length and 2-3 nm in diameter, undergo self-assembly, resulting in the formation of larger supramolecular fibre structures with diameters of ∼500 nm (Bai et al., 2013; Shoulders and Raines, 2009). The concentric and periodic stacking pattern of collagen molecules facilitates plate-shaped nanocrystal (10-20 nm in length and 2-3 nm in width) interactions, enabling further arrangement of the larger mineralised fibres into macro-level tissue structures. Polarisation-resolved second-harmonic generation (p-SHG) is a powerful non-destructive multiphoton modality that has previously been utilised for the study of collagen architecture and fibrillar orientation (Stoller et al., 2002). This technique has been used to inform and assess the pathological consequence of disease on extracellular matrix (ECM) structure and conformation in a variety of cancers (Campbell et al., 2018; Chen et al., 2012; Tilbury et al., 2014b; Tokarz et al., 2019), fibrosis (James et al., 2019; Johnson et al., 2020 preprint; Lin et al., 2013; Rouède et al., 2017; Tilbury et al., 2014a) and in skin (Ducourthial et al., 2019; Fukushima et al., 2018; Su et al., 2009). Furthermore, p-SHG has been applied to investigate collagenous alterations that accompany ageing (Van Gulick et al., 2019), wound repair and scar formation (Mostaço-Guidolin et al., 2017). In a number of skeletal studies, second-harmonic generation (SHG) has also been used to (1) visualise woven bone formation during fracture repair (Park et al., 2019), (2) elucidate the effects of mechanical loading on collagen formation (Mansfield et al., 2019; Pinsard et al., 2019), and (3) differentiate between normal and OI bone tissue (Nadiarnykh et al., 2007). To date, however, multiscale quantitative analyses of collagen organisation, fibre alignment or anisotropy combined with the assessment of bone mineral composition within pathological bone have not been studied.

**Fig. 1.**
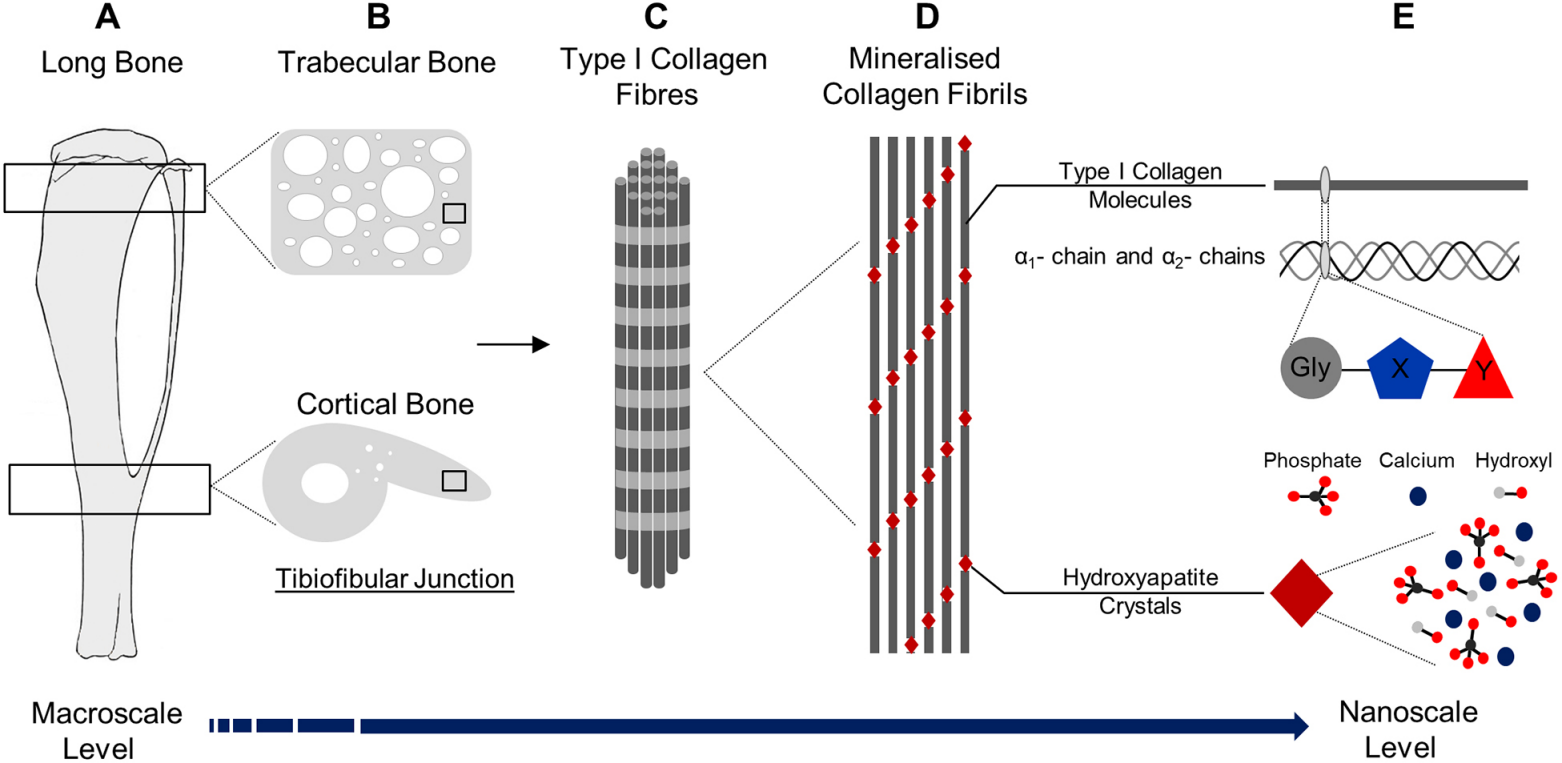
**Multiscale organisational hierarchy of bone.** (A-E) The macroscopic murine long bones (A) are organised into several lower microstructural levels including trabecular bone found at the epiphyses and cortical bone, hosting the tibiofibular junction, in the mid-diaphysis (B). Within this exists a lower hierarchical level comprising extracellular matrix, containing type I collagen fibres (C) formed of bundles of fibrils (D) that are mineralised with hydroxyapatite (HA) crystals. The ultrastructural arrangement of these components is observed at the nanoscale level and constitutes molecular moieties, such as amino acid residues in triple helical type I collagen molecules in addition to inorganic phosphate, calcium ions and hydroxyl groups in HA (E).

Vascularisation of the collagen matrix is a requirement for mineralisation and is driven, in part, by angiogenic factors produced by matrix-forming OBs, including vascular endothelial growth factor (VEGF; also known as VEGFA) (Hu and Olsen, 2016; Liu et al., 2012). We recently reported that loss of *Vegf* in osteocalcin-expressing OBs (OcnVEGFKO) promotes a poorly mineralised, yet highly vascularised, bone patho-phenotype that is pronounced in males (Goring et al., 2019). *In vitro* studies confirmed that this role of VEGF was, in part, mediated via autocrine signalling in male OBs, which resulted in compromised bone maturation. Murine long bones exist as compartmentalised structures with profound hierarchical arrangement observable on multiple levels. At the macroscale, trabecular and cortical structures (Fig. 1A,B) are visible and are distinct by their epiphyseal or diaphyseal localisation, respectively. Despite these differences in spatial organisation, nano-molecular type I collagen and HA crystals, formed as a product of OB differentiation, constitute the majority of the mineralised ECM throughout the bone (Florencio-Silva et al., 2015) (Fig. 1C-E). In order to investigate the resultant effects of VEGF depletion at the macro- and nanoscale, we combined p-SHG with Raman spectroscopy to characterise the spatial deviations in ECM organisation and composition. Linked with this multiscale analysis, we examined the potential mechanisms underlying the uncovered matrix signatures through the assessment of gene regulation *in vitro* following *Vegf* deletion in primary murine male and female OBs.

## RESULTS

### p-SHG imaging reveals alterations in collagen fibre number following VEGF deletion in males

**Fig. 2.**
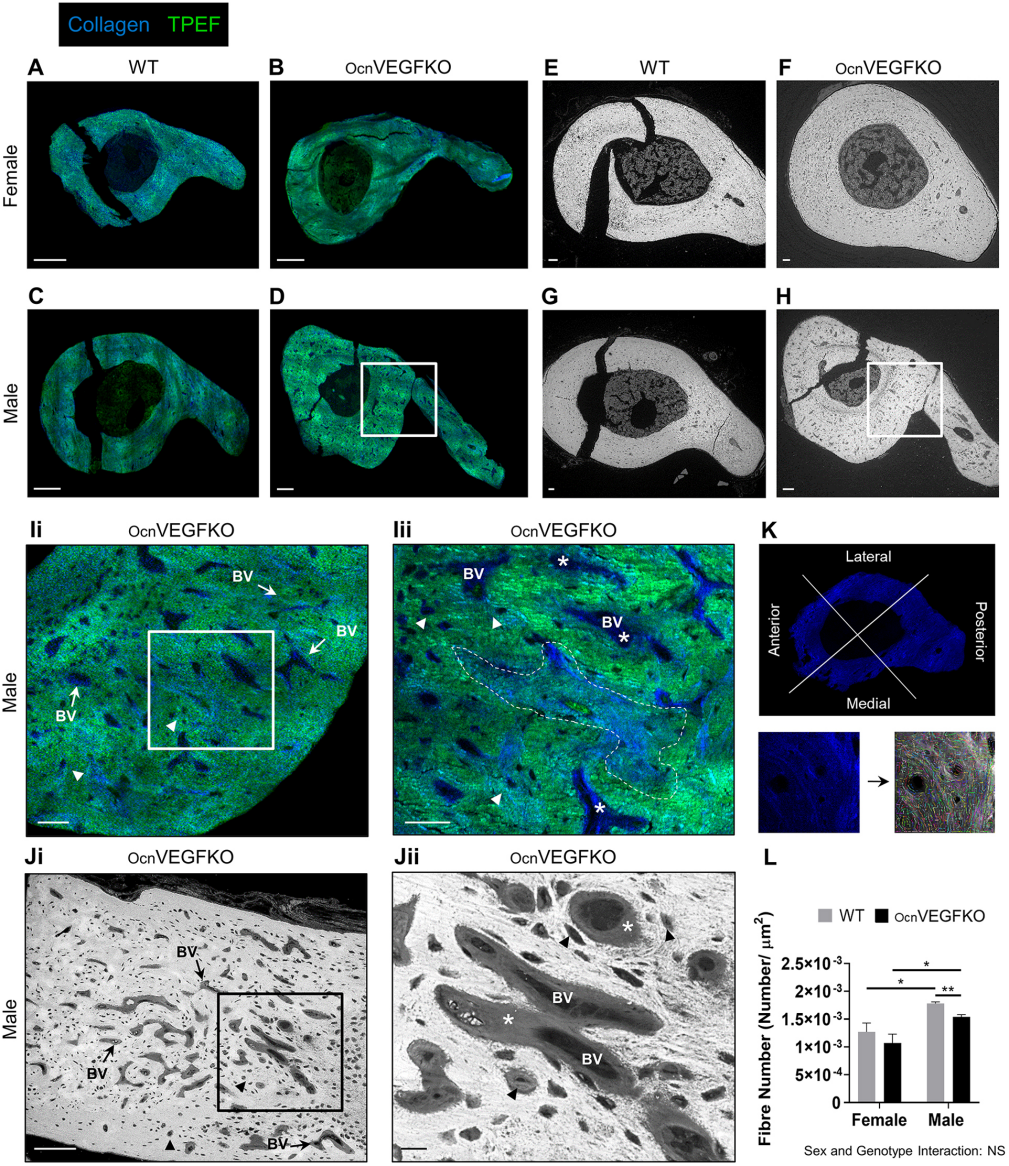
**Sexually dimorphic deficiencies in extracellular matrix** **(ECM)** **organisation observed at the macroscale.** (A-D) Tibiofibular junction (TFJ) bone sections from female wild-type (WT) (A), female osteocalcin-specific *Vegf* knockout (OcnVEGFKO) (B), male WT (C) and male OcnVEGFKO (D) animals were imaged using a custom-built multiphoton microscope enabling simultaneous polarisation-resolved second-harmonic generation (p-SHG) and two-photon excitation fluorescence (TPEF). Collagen fibres in the p-SHG channel are shown in blue; the TPEF channel highlights mineralised matrix, shown in green. (E-H) Corresponding backscattered electron scanning electron microscopy (BSE-SEM) images show vascular canals and osteocyte lacunae across sections from the same animals. (Ii-Jii) Zoomed p-SHG/TPEF (Ii) and BSE-SEM (Ji) images of the posterior cortex in male OcnVEGFKO bone, defined by boxes in D and H, respectively, are shown. Further zoomed regions (Iii,Jii) are defined by the boxes in Ii and Ji, respectively. Arrows point to abnormal vascular canals (BV, blood vessels). Arrowheads point to osteocyte lacunae. The area within the dashed line (Iii) and asterisks (Iii,Jii) show regions of unmineralised osteoid-like matrix surrounding vascular canals and osteocyte lacunae. (K) Stitched TFJ p-SHG images were manually segmented into the anterior, lateral, medial and posterior quadrants prior to localised analysis of fibre number confined to the posterior region using CT-FIRE. (L) Image quantification revealed sex differences in collagen fibre number between WT and OcnVEGFKO animals, in addition to sex-specific reductions following OcnVEGFKO. Data are presented as mean normalised fibre number±s.e.m. from *n*=3 female and *n*=3 male bone sections per genotype. Statistical significance between groups was assessed using paired Student's *t*-test (*P<0.05, **P<0.01; NS, not significant). Interaction between sex and genotype was assessed using two-way ANOVA. Scale bars: 200 µm (A-D), 100 µm (F,G,Ji), 50 µm (Ii,Iii) and 20 µm (E,H,Jii).

Dual-channel p-SHG and two-photon excitation fluorescence (TPEF) imaging could detect the distribution of collagen fibres (p-SHG; blue) across the mineralised ECM (TPEF; green) at the tibiofibular junction (TFJ) of female wild-type (WT; Fig. 2A), female OcnVEGFKO (Fig. 2B), male WT (Fig. 2C) and male OcnVEGFKO (Fig. 2D) bone sections. Parallel backscattered electron scanning electron microscopy (BSE-SEM) images of these bones showed organised and mineral-embedded ECM displaying typical bone microstructure, comprised of vascular canals and osteocyte lacunae (Fig. 2E-H). In male OcnVEGFKO animals, extensive collagen matrix disorganisation visible in p-SHG/TPEF images (Fig. 2D) around blood vessel canals (Fig. 2Ii,Iii, BV, arrows) and osteocyte lacunae (Fig. 2Ii,Iii, arrowheads) appeared in the posterior cortex as patches of unmineralised osteoid in iodine-stained BSE-SEM images (Fig. 2H,Ji,Jii, asterisks). Moreover, additional patches of low p-SHG signal intensity were observed throughout the bone cortex (Fig. 2Iii, area within the dashed line).

The cortical bone microstructure at the TFJ exhibits regional heterogeneity, with the highly vascularised posterior cortex exclusively exhibiting age-related changes in vascularity (Núñez et al., 2018). In line with this, we performed localised collagen fibre measurements in the posterior compartment following manual segmentation of the p-SHG TFJ images (Fig. 2K). Males were observed to have a significantly greater number of collagen fibres than females in both WT and OcnVEGFKO animals (Fig. 2L; Fig. S1A-D). No significant differences in the number of fibres were observed following OcnVEGFKO in females; however, fibre number/µm^2^ was significantly reduced in OcnVEGFKO males compared to their respective WT controls. No significant interaction between sex and genotype on fibre number was reported following two-way analysis of variance (ANOVA).

### Spatial anisotropy of fibrillar collagen following loss of VEGF in bone is sex specific

**Fig. 3.**
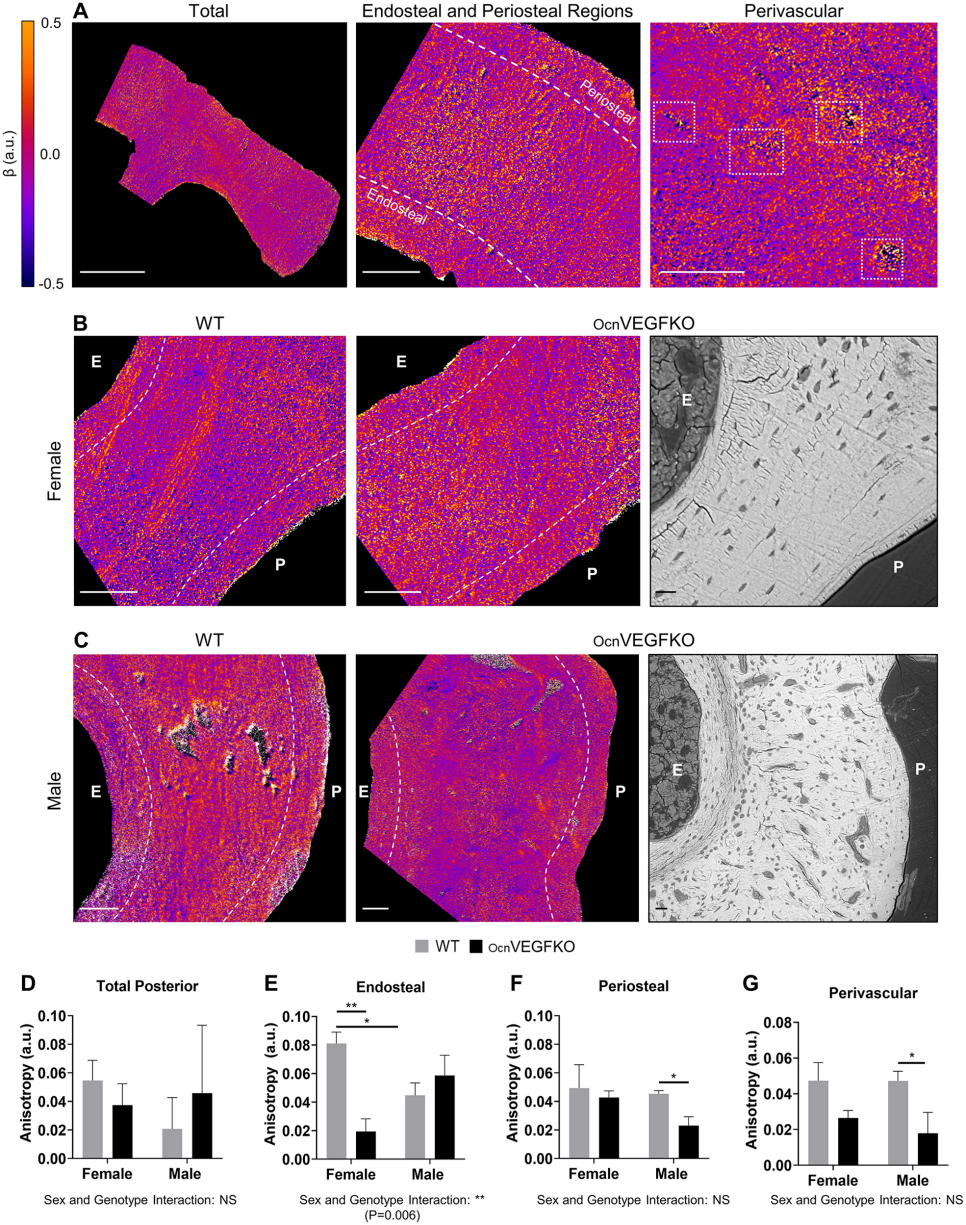
**SHG polarisation anisotropy identifies morphological disorder in collagen fibril alignment in the posterior region of the TFJ following OcnVEGFKO.** (A) Polarisation-resolved anisotropy analysis was performed on the total posterior region (left), on the endosteal and periosteal areas (middle; dashed lines) and around the vascular canals (right; boxed). (B,C) Zoomed SHG polarisation anisotropy images of the posterior region of the TFJ are shown for female WT (B; left), female OcnVEGFKO (B; middle), male WT (C; left) and male OcnVEGFKO (C; middle) bones around the endosteal (depicted as E) and periosteal (depicted as P) regions. BSE-SEM images show endosteal and periosteal differences following OcnVEGFKO in females (B; right) and males (C; right). (D-G) The anisotropy parameter, β, calculated pixel-wise across the whole posterior region (D), around the endosteal (E) and periosteal (F) regions, and around blood vessel canals (G) highlighted sex differences in local fibril organisation in WT and OcnVEGFKO animals and following VEGF depletion in OcnVEGFKO animals versus WT controls. Data are presented as mean anisotropy value±s.e.m. from *n*=3 female and *n*=3 male bone sections per genotype. Statistical significance between groups was assessed using paired Student's *t*-test (*P<0.05, **P<0.01; NS, not significant). Interaction between sex and genotype was assessed using two-way ANOVA. Scale bars: 200 µm (A, left), 100 µm (B, right), 50 µm (A, middle and right; B,C, left and middle) and 10 µm (C, right). a.u., arbitrary units.

Anisotropy determined from p-SHG images provided a quantitative measure of fibril alignment. This allowed us to assess the spatial organisation of the collagen fibrils within the ECM of OcnVEGFKO animals. To investigate whether the alterations to fibrillar alignment were responsible for the matrix-level abnormalities observed as a result of OcnVEGFKO, the mean anisotropy parameter, β, was calculated pixel-wise across the posterior region (Fig. 3A). The value of this parameter is variable, between −0.5 and 1 (Campagnola et al., 2002), with these values signifying physical anisotropy or complete fibrillar alignment relative to the excitation laser polarisation with respect to their direction. In the opposing case, isotropic or random fibrillar alignment occurs exclusively when β=0. In the current study, values of β within the range of 0.5 to −0.5 were observed (Fig. 3A).

In the p-SHG anisotropy images, no significant differences in anisotropy, β, were observed across the total posterior region between the sexes and genotypes (Fig. 3A,D). BSE-SEM of iodine-stained sections of OcnVEGFKO bones highlighted distinctive and compartmentalised alterations in the ECM organisation, particularly in males (Fig. 3B,C, right). We subsequently undertook further analysis within this region of interest (ROI), including the endosteal and periosteal areas (Fig. 3A, middle) and around the vascular canals (Fig. 3A, right). Sex differences in fibril alignment were only apparent in WT animals in the endosteal regions, with fibrils being significantly more anisotropic in females versus males (1.81-fold, Fig. 3E). In addition, within this region, loss of VEGF in female OcnVEGFKO animals resulted in a reduction in anisotropy (−4.19-fold) versus WT alone (Fig. 3B,E), which was not evident in either the periosteal or perivascular regions (Fig. 3B,F,G). In contrast, in the periosteal and perivascular regions, OcnVEGFKO significantly decreased anisotropy in males by 1.97-fold and 2.64-fold, respectively (Fig. 3C,F,G), which may be contributing to the observable divergence in matrix organisation between the sexes throughout the posterior cortex. To assess the interaction between sex and genotype on anisotropy for each ROI, two-way ANOVA was conducted. A significant interaction between sex and genotype was exclusively reported in the endosteal regions (Fig. 3E), with no additional interactions detected for the total posterior, periosteal or perivascular regions (Fig. 3D,F,G, respectively).

### Sexual dimorphism in molecular ECM signatures following loss of VEGF identified by Raman spectroscopy

**Fig. 4.**
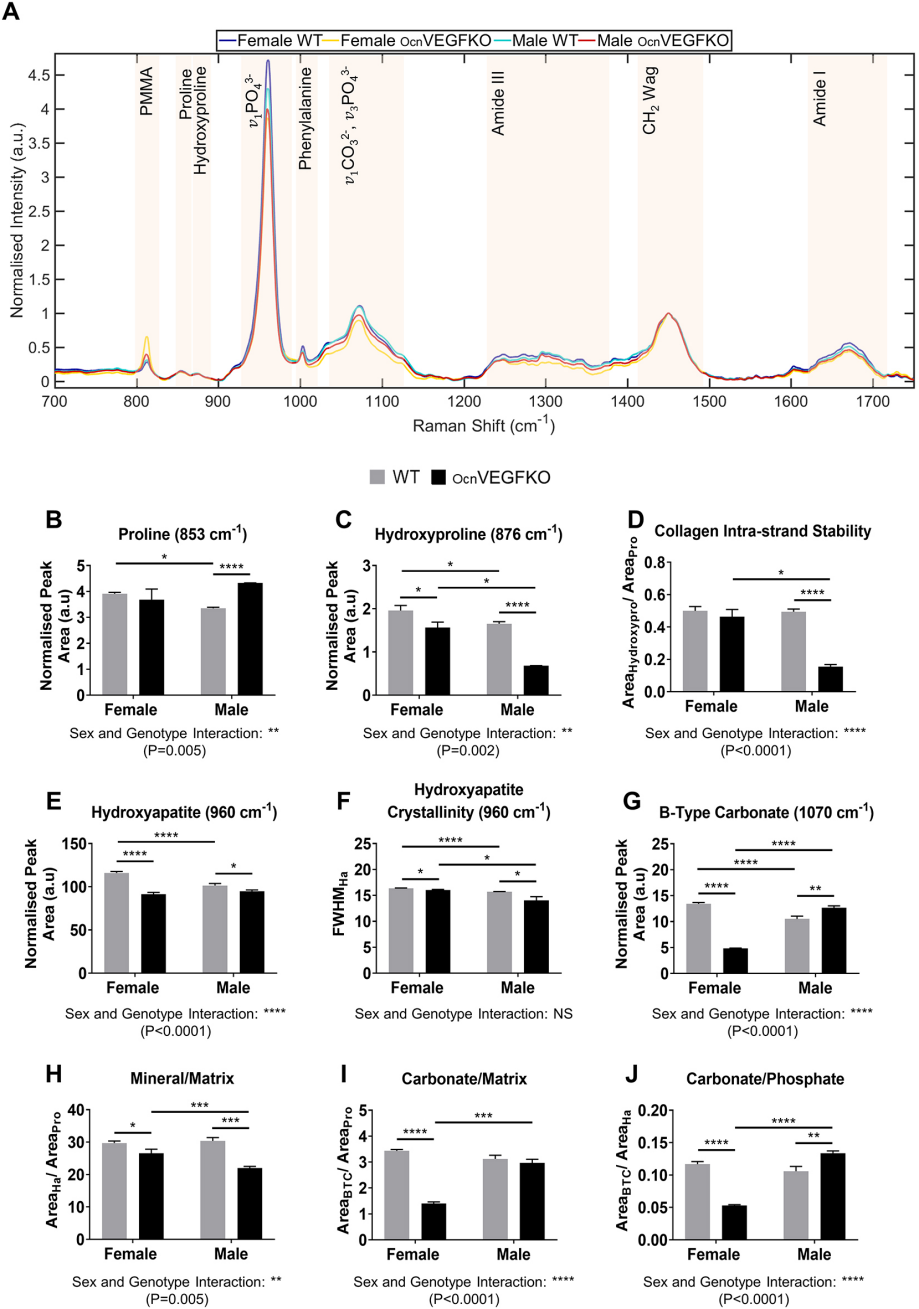
**Raman spectroscopy identifies correlative sexual dimorphism in bone matrix signatures.** (A) Deviations in class means of normalised spectra (*n*=75 spectra per group) were observed in Raman bands associated with the organic bone ECM (proline, 853 cm^−1^; hydroxyproline, 876 cm^−1^; amide III region, 1242 cm^−1^; CH_2_ wag, 1450 cm^−1^; amide I, 1660 cm^−1^) and mineral components (HA, v_1_PO_4_^3−^, 960 cm^−1^; B-type carbonate, 1070 cm^−1^) across the sexes between genotypes. (B,C) Spectral deconvolution of Raman spectra revealed sex differences in components associated with mature type I collagen (proline, B; hydroxyproline, C) in WT and OcnVEGFKO animals, in addition to sex-specific VEGF effects in OcnVEGFKO animals versus WTs. (D) These changes resulted in a reduction in the collagen intra-strand stability, with sex differences detected in OcnVEGFKO animals, and in male OcnVEGFKO bones versus WT control bones. (E-G) Diversity in the levels of HA (E), HA crystallinity (F) and B-type carbonate (G) were also detected across the sexes and genotypes. (H-J) The dimorphic modifications to the collagen-specific and mineral species, emphasised by the mineral/matrix ratio (H), the carbonate/matrix ratio (I) and the carbonate/phosphate ratio (J), highlighted additional sex differences between WT and OcnVEGFKO animals and the sex-specific effects of VEGF loss in OcnVEGFKO animals versus WT controls. Data are presented as the mean normalised peak area or full-width half-maximum (FWHM)±s.e.m. from *n*=3 female and *n*=3 male bone sections per genotype. Statistical significance between groups was assessed using paired Student's *t*-test (*P<0.05, **P<0.01, ***P<0.001, ****P<0.0001; NS, not significant). Interaction between sex and genotype was assessed using two-way ANOVA.

Raman spectroscopy was conducted to assess whether the visible defects in the organisation of the bone matrix collagen, in the posterior cortex, were driven in part by alterations in composition following VEGF loss. Spectral deviations in collagen-specific species, including proline (853 cm^−1^) and hydroxyproline (876 cm^−1^), HA mineralisation (v_1_PO_4_^3−^, 960 cm^−1^), HA crystallinity [full-width half maximum (FWHM) at 960 cm^−1^] and B-type carbonate (1070 cm^−1^), in addition to noted fluctuations in amide III (1242 cm^−1^) and amide I (1660 cm^−1^), were detectable across animals (Fig. 4A). To quantify the basal sex differences in WT and OcnVEGFKO animals, in addition to the effect of VEGF loss in OcnVEGFKO animals on type I collagen, the 852 cm^−1^ and 876 cm^−1^ bands for proline and hydroxyproline, respectively, were selected, owing to their respective frequency in the glycine-X-Y repeat of type I collagen (Shoulders and Raines, 2009). Proline levels were 1.17-fold higher in female versus male WT animals (Fig. 4B). Additionally, OcnVEGFKO had no effect on proline levels in females; however, a significant elevation was observed in OcnVEGFKO males (1.29-fold) compared to their respective WT controls. Sex was also observed to influence hydroxyproline, with the levels in female WT and OcnVEGFKO posterior cortices being 1.19-fold and 2.30-fold higher than in the male WT and OcnVEGFKO posterior cortices, respectively (Fig. 4C). A small, but significant, reduction in hydroxyproline levels was induced following VEGF loss in females (–1.25-fold), with considerably larger reductions (−2.43-fold) detected in OcnVEGFKO males. The imbalance in the levels of these collagen-specific components can lead to modifications in the stability of the intra-strand links in type I collagen molecules. Calculated by the area ratio of the hydroxyproline to proline bands in Raman spectra, the only detectable sex difference in collagen intra-strand stability was between OcnVEGFKO animals, with stability being 2.97-fold higher in females versus males (Fig. 4D). The intra-strand stability was not affected by OcnVEGFKO in females; however, a 3.06-fold reduction was noted in male OcnVEGFKO animals versus the respective WTs.

Upon the inspection of mineral components, including HA and carbonate species, further sex differences were apparent. The levels of HA detected in the posterior cortex were greater (1.14-fold, Fig. 4E) and more crystalline (1.04-fold, Fig. 4F) in female than in male WTs. Although no differences in HA levels between male and female OcnVEGFKO animals were detected, VEGF loss translated in the reduction of HA (−1.27-fold, Fig. 4E) and loss of crystallinity (−1.02-fold, Fig. 4F) in female OcnVEGFKO animals versus their respective controls. In contrast, a 1.07-fold reduction in HA (Fig. 4E) and a 1.12-fold reduction in crystallinity (Fig. 4F) was detected in male OcnVEGFKO animals versus their WT counterparts. Throughout the dynamic process of apatite maturation, phosphate ions within the mineral lattice are susceptible to ionic substitution, with the most common being that for carbonate ions. Therefore, we examined the effect of sex and the consequential effect of VEGF depletion on the presence of such carbonate species within the ECM. In WT and OcnVEGFKO animals, sex was observed to affect the levels of B-type carbonate divergently. In WTs, carbonate levels were 1.28-fold higher in females than in males, whereas in OcnVEGFKO animals, the levels of carbonate were 2.64-fold higher in males than in females (Fig. 4G). Similar divergence in carbonate species levels was evident following OcnVEGFKO. In females, OcnVEGFKO resulted in a significant, 2.79-fold reduction in B-type carbonate levels, whereas, in contrast, a 1.21-fold increase in carbonate presence was detected in male OcnVEGFKO animals.

These oppositional changes in the bone ECM composition were further reinforced by the mineral/matrix ratio, the carbonate/matrix ratio and the carbonate/phosphate ratio, which are typically used to report bone quality and predict changes in mechanical competence (Mandair and Morris, 2015). Sex in WT animals had no effect on the mineral/matrix ratio, commonly used to define matrix maturity, whereas a 1.21-fold reduction was observed in male versus female OcnVEGFKO animals. VEGF loss was also observed to implicate the matrix maturity, with 1.38-fold reductions evident in male OcnVEGFKO animals, and smaller reductions observed in female OcnVEGFKO animals, compared to WTs (−1.12-fold, Fig. 4H). Interestingly, the carbonate/matrix ratio, typically applied to estimate the extent of carbonate mineralisation and an indirect predictor of fracture risk, was similarly affected by sex. Like with the mineral/matrix ratio, sex had no effect in WT animals; however, the potential predisposition for fracture was 2.12-fold greater in male versus female OcnVEGFKO animals (Fig. 4I). Following OcnVEGFKO, the carbonate/matrix ratio in females was significantly reduced by 2.45-fold. In contrast, no significant differences in this ratio were detected in male OcnVEGFKO bone compared to WT bone. These changes were linked to the ionic substitution of carbonate for phosphate within the ECM as described by the carbonate/phosphate ratio. Mirroring the trends observed for the carbonate/matrix ratio, the carbonate/phosphate ratio was elevated by 2.53-fold in male versus female OcnVEGFKO animals, whereas no differences were detected between male and female WT animals. VEGF loss was observed to significantly reduce this ratio by 2.4-fold in OcnVEGFKO females, but increase it by 1.18-fold in OcnVEGFKO males, compared to their WT counterparts (Fig. 4J). Two-way ANOVA reported a significant interaction between sex and genotype for all Raman bands and ratios, with the exception of HA crystallinity, for which no interaction between sex and genotype was detected (Fig. 4B-J).

### Regulation of ECM genes in male and female OBs following *Vegf* deletion

**Fig. 5.**
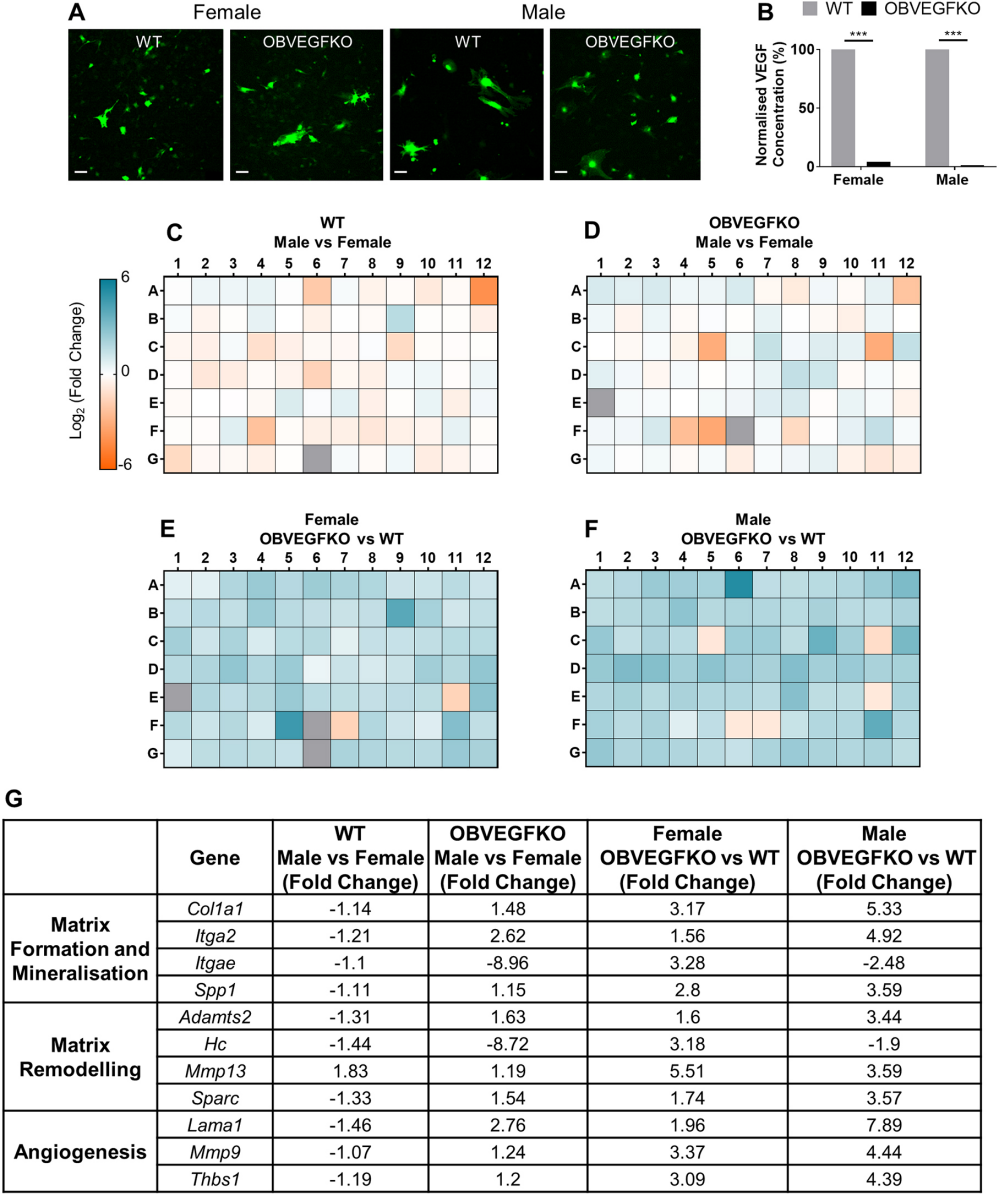
**Deletion of VEGF in osteoblasts (OBs) *in vitro* drives alterations in gene expression associated with ECM organisation and composition.** (A) Representative fluorescence images of primary murine male and female OBs transduced with adenovirus expressing GFP (WT) or expressing Cre-recombinase (OBVEGFKO) *in vitro* are shown. (B) Knockdown was confirmed by VEGF ELISA of conditioned media. (C-F) mRNA expression of 84 genes associated with the ECM and adhesion molecules, screened by qPCR array (*n*=1 cDNA), are displayed as heatmap diagrams of log-transformed fold change in relative expression levels. Heatmaps highlight sex differences between WT cells (C) and OBVEGFKO cells (D) in addition to the effect of OBVEGFKO in female (E) and male (F) *Vegf*-deficient OBs versus *Vegf*-expressing WTs. (G) Table summarising the effect of OBVEGFKO on the expression of key genes expressed as fold change. Statistical significance between groups was assessed using paired Student's *t*-test (***P<0.001). Scale bars: 100 μm.

To elucidate the potential transcriptional regulators underlying the sexual dimorphism of the ECM, *Vegf* expression was deleted in OBs extracted from male and female mice (denoted as OBVEGFKO; Fig. 5A) *in vitro* prior to gene expression analyses using an ECM quantitative PCR (qPCR) array. Successful loss of VEGF protein release was confirmed by VEGF enzyme-linked immunosorbent assay (ELISA) of culture media, in both male (98.6%) and female (96.1%) *Vegf-*deficient OBVEGFKO cells versus WT cells (Fig. 5B).

Male WT OBs expressed lower levels of mRNA transcripts for genes associated with ECM formation and mineralisation, including type I collagen (*Col1a1*, −1.14-fold), the ECM-binding integrin α2 and αE subunits (*Itga2* and *Itgae*, −1.21 and −1.1-fold, respectively) and secreted phosphoprotein 1/osteopontin (*Spp1*, −1.11-fold), versus female WT OBs (Fig. 5C,G). Such trends were not apparent in OBVEGFKO cultures, as the expression of *Col1a1*, *Itga2* and *Spp1* was upregulated by 1.48-fold, 2.62-fold and 1.15-fold, respectively, in male versus female OBVEGFKO cells, with the exception of *Itgae*, for which expression was downregulated by 8.86-fold in male versus female OBVEGFKO cells (Fig. 5D,F). In female OBs, following *Vegf* deletion, the mRNA transcript levels of *Col1a1*, *Itga2* and *Spp1* were upregulated (3.17-fold, 1.56-fold and 2.99-fold, respectively) versus the levels in WT (Fig. 5E,G). These transcriptional changes induced by OBVEGFKO were further enhanced in male cells, with greater upregulation of mRNA expression observed versus WT controls (*Col1a1*, 5.33-fold; *Itga2*, 4.92-fold; *Spp1*, 3.59-fold; Fig. 5F,G). Interestingly *Itgae* expression appeared to be dimorphically regulated by *Vegf*, as transcript levels were upregulated by 3.28-fold in female OBVEGFKO cells but downregulated by 2.48-fold in male OBVEGFKO cells versus their respective WTs (Fig. 5G).

Interrogation of the genes involved in matrix remodelling revealed that the male WT OBs also expressed lower basal levels of A disintegrin-like and metallopeptidase with thrombospondin type 1 motif 2 (*Adamts2*, −1.31-fold), haemolytic complement (*Hc*, −1.44-fold) and secreted acidic cysteine rich glycoprotein/osteonectin (*Sparc*, −1.33-fold) than females, whereas matrix metallopeptidase 13 (*Mmp13*) mRNA levels were 1.83-fold higher in male versus female WT cells. Contrasting with this, OBVEGFKO was observed to induce differential effects on the expression of these genes, with male OBVEGFKO cells exhibiting higher levels of *Adamts2* (1.63-fold), *Mmp13* (1.19-fold) and *Sparc* (1.54-fold) expression than female OBVEGFKO cells. Similarly to *Itgae*, *Hc* mRNA expression was downregulated by 8.72-fold in male versus female OBVEGFKO cells. Following the loss of *Vegf*, *Hc* expression levels were dimorphically influenced by OBVEGFKO; upregulated by 3.18-fold in female OBVEGFKO versus WT cells but downregulated by 1.9-fold in male OBVEGFKO versus WT cells. *Adamts2* (1.6-fold and 3.44-fold), *Mmp13* (5.51-fold and 3.59-fold) and *Sparc* (1.74-fold and 3.57-fold) expression levels were upregulated in both male and female OBVEGFKO cultures, compared to WT controls.

Given that OB-derived VEGF is an important regulator of angiogenesis, mediated via paracrine signalling to endothelial cells, we additionally investigated the effect of sex in *Vegf*-expressing and -deficient cells, in addition to the effect of *Vegf* depletion in male and female OB cultures, on the expression of genes associated with autocrine control of pro-angiogenic bone matrix formation. The expression of genes encoding laminin subunit α1 (*Lama1*), matrix metallopeptidase 9 (*Mmp9*) and thrombospondin 1 (*Thbs1*) was lower in male versus female WT cells (−1.48-fold, −1.07-fold and −1.19-fold, respectively) but higher in male versus female OBVEGFKO cells (2.76-fold, 1.24-fold and 1.2-fold, respectively). Interestingly, OBVEGFKO resulted in the upregulation of *Lama1* (1.96-fold), *Mmp9* (3.37-fold) and *Thbs1* (3.09-fold) in female cells versus their respective WTs. To a similar extent, *Vegf* loss translated in the extensive upregulation of these genes (7.89-fold, 4.44-fold and 4.39-fold, respectively) in male OBVEGFKO cells in comparison to their WT controls, with higher overall expression changes observed following OBVEGFKO in males than in females (Fig. 5E-G).

## DISCUSSION

The combination of multimodal p-SHG and TPEF imaging with Raman spectroscopy has revealed that the compositional abnormalities in pathological bone are associated with structural alterations in collagen conformation and matrix mineralisation. Uniquely, these techniques have enabled the in-depth assessment and characterisation of nanoscale sex differences in matrix composition following the loss of VEGF. Previous studies linked the loss of VEGF to bone pathology characterised by a profound osteoporotic phenotype present following the deletion of *Vegf* in early and late-stage OBs (Goring et al., 2019; Liu et al., 2012). Furthermore, clinical reports have also identified that the loss of VEGF is associated with the degenerative bone loss seen in osteoporosis (Senel et al., 2013).

Label-free p-SHG imaging of calcified tibial sections from OcnVEGFKO animals identified a reduction in the number of collagen fibres exclusively in the male bones, which were localised to the highly vascularised posterior region of the TFJ. Anisotropy in p-SHG signals revealed variations in fibrillar collagen organisation and highlighted distinctive, sex-specific alterations in the endosteal compartment of the posterior cortex in female OcnVEGFKO animals and in periosteal and perivascular regions in male OcnVEGFKO animals. Raman spectroscopy further identified that these localised deviations in the arrangement of the collagenous matrix following VEGF loss were associated with divergence in the levels of nano-molecular ECM components. Specifically, in the male OcnVEGFKO bone alone, deviation in the ratio of hydroxyproline to proline indicated further dimorphism in fibrillary collagen conformation and integrity at the nanoscale level. Although the collagen content of bone is an important determinant of toughness and resistance to fracture (Boskey et al., 1999; Nyman and Makowski, 2012; Unal et al., 2018), the strength of bone is largely dictated by its mineral phase (Burstein et al., 1975; Morris and Mandair, 2011; Viguet-Carrin et al., 2006). In both male and female bone, the loss of VEGF in OcnVEGFKO animals resulted in reductions in the levels of HA. These trends were extended to the mineral/matrix ratio, used to assess the extent of matrix mineralisation in bone (McCreadie et al., 2006). These dimorphic matrix signatures were linked to a number of genes involved in matrix arrangement that exhibited divergent regulation following the deletion of *Vegf* in male and female OBs *in vitro*. As the sexual dimorphism observed in fibrillary collagen arrangement and ECM composition was apparent at the nanoscale level, we report novel findings and demonstrate the robust analytical capabilities of complementary techniques that can link the molecular manifestations to the sex-specific deficiencies in matrix organisation observed at the macroscale level.

The material properties of bone are determined by both the mineral and organic constituents of the ECM. Thus, bone strength and flexibility, independent of bone mass, are ultimately compromised by deficiencies in composition. Previous investigations have reported that the collagen fibrils in cortical bone adopt a preferential alignment with anisotropic fibrils bearing alignment transverse to the long axis, enabling resistance to compressive forces, whereas those orientated longitudinally confer enhanced resistance to tensile forces (Ascenzi and Benvenuti, 1986; Augat and Schorlemmer, 2006; Li et al., 2013; Martin and Boardman, 1993; Morgan et al., 2018; Simkin and Robin, 1974). We predict that the reductions in the number of collagen fibres and localised anisotropy around the periosteal and perivascular regions of the posterior cortex in male OcnVEGFKO bone may contribute to a sex-specific divergence in mechanical properties. This idea is supported by BSE-SEM imaging of the male OcnVEGFKO TFJ cortex, which highlighted extensive areas of unmineralised osteoid densely confined around the vascular canals that coalesced with areas of diffuse p-SHG signal. A principal idea surrounding anisotropy in bone is that the orientation of collagen fibres and nucleating mineral crystals bears alignment to the direction of bone growth (Wang et al., 2012). Therefore, zonal alterations to fibril anisotropy observed in the OcnVEGFKO bone may reveal developmental adaptation confined to specific areas, such as abnormal vascular canal growth within the bone cortex. As a consequence, this may primarily result in irregularities in collagen organisation that lead to further secondary deficiencies in matrix mineralisation.

Aside from structural organisation, the chemical composition of bone is an important contributor of bone quality and strength (Mandair and Morris, 2015; Morris and Mandair, 2011). As the deposited collagen functions as a scaffold for bone mineralisation, we investigated whether dimorphic changes in the levels of collagen-specific species following OcnVEGFKO are related to the defective mineralisation phenotype. Type I collagen molecules are structurally composed of a triple amino acid repeat (Gly-X-Y), with the most abundant amino acids in the X and Y position being proline (28%) and hydroxyproline (35%), representing the most common triplicate motif (Ramshaw et al., 1998). Using Raman spectroscopy, we showed that elevations in proline and reductions in hydroxyproline were present in OcnVEGFKO versus WT males, whereas only hydroxyproline levels were reduced in OcnVEGFKO versus WT females. The transition from proline to hydroxyproline in collagen molecules occurs as a post-translational event and is critical for the folding of the collagen triple helix into fibrillary structures, enhancing internal hydrogen bonding between adjacent polypeptide chains (Bansal et al., 1975). As a result, the imbalances in hydroxyproline to proline levels assessed in the collagen intra-strand stability ratio (Olejnik et al., 2016; Sharma et al., 2020) indicated that severe compromise in the conformational stability of the collagen fibrils only occurs in the male OcnVEGFKOs. We also noted reductions in the levels of HA and mineral crystallinity in both male and female OcnVEGFKO bones, alongside sexually dimorphic deviations in carbonate levels. HA crystals in bone exist as nanocomposite platelet structures that can readily accommodate ionic substitution of anionic phosphate at the PO_4_^3−^ sites (B-site), with the most common replacement being that with carbonate [B-type (Madupalli et al., 2017; Morris and Mandair, 2011)]. The effect of carbonate substitution within the apatite lattice is relatively well documented, with studies using vibrational spectroscopies and X-ray diffraction reporting inverse relationships of carbonate content in bone with mineral crystallinity (McElderry et al., 2013; Pleshko et al., 1991). In line with our findings, reductions in B-type carbonate levels in the female OcnVEGFKO cortex coincided with relatively small reductions in the HA crystallinity. In comparison, in males, carbonate levels were significantly increased and greater reductions in crystallinity were observed following OcnVEGFKO, strongly indicating that biomechanical deficit and fracture susceptibility may occur in a sex-specific manner as a consequence of the VEGF loss (Mandair and Morris, 2015; Morris and Mandair, 2011; Yerramshetty and Akkus, 2008).

Bone strength, determined by the acquisition of peak bone mass, is regulated during puberty and determines the sex differences in bone length, BMD, geometry and microarchitecture that persist into adulthood and old age. The sexually dimorphic control of the material properties of bone, however, continues to be poorly understood, and whether diversity in genetic regulators of collagen formation and arrangement translate in regionalised differences in cortical bone pre-pubertally remains an intriguing possibility. Supporting our findings in mice, age-related and regional sex differences in collagen fibre orientation have been reported in humans (Goldman et al., 2003). Furthermore, sexual dimorphism in collagen production was recently reported in the skin of mice, as a result of divergence in the expression of type I collagen mRNA during postnatal skin development at the onset of puberty (Arai et al., 2017). In bone, mutations in genes encoding type I collagen result in OI, a developmental pathology characterised by a distinctive and extremely fragile skeletal phenotype (Bai et al., 2013; Chen et al., 2012; Stoller et al., 2002; Yao et al., 2013). Previous multiscale analyses of a murine OI model utilised a combination of micro-computed tomography and three-point mechanical bending with Raman spectroscopy to reveal sex-specific trends in geometrical, mechanical and compositional parameters (Yao et al., 2013). Sexual dimorphism was reported in cortical and trabecular bone morphology, with male OI mice having greater bone mass and, as a result, increased biomechanical strength. However, associated differences were not evident in hydroxyproline content, or in the mineral/matrix, the carbonate/matrix or the carbonate/phosphate ratio between male and female OI animals. This result was somewhat surprising as the authors concluded that mineral and matrix components in OI bone do not exhibit the same sex dependency as the induced geometrical and biomechanical deficits in the tibial cortex of their murine model. An alternative explanation, and possible limitation, is that the Raman spectra were averaged across the anterior, lateral, medial and posterior compartments, causing potential dilution of any detectable regionalised effects.

The involvement of sex hormones in controlling the sex-specific deviations in matrix composition is an interesting question. Although the role of oestrogen in regulating collagen production in skin is well investigated (Arai et al., 2017; Brincat et al., 1987; Markova et al., 2004; Son et al., 2005), controversy surrounding the response of osteoblastic cells to sex steroids during bone collagen synthesis remains (Canalis and Raisz, 1978; Keeting et al., 1991; Majeska et al., 1994; Wiren et al., 2008). In the present study, successful *in vitro* culture of male and female OBs was achieved in the absence of circulating hormones. In addition, the expression of both oestrogen and androgen receptors in OBs derived from male and female mice were comparable (Goring et al., 2019). Deletion of *Vegf* in male and female OBs influenced the expression of genes associated with ECM formation and arrangement, confirming a potential intracrine and/or autocrine role of VEGF signalling in bone. Consistent with a reduction in mineralisation in the male OcnVEGFKOs, we report elevated expression of genes associated with matrix remodelling and angiogenesis in male *Vegf*-deficient OBs. Furthermore, we observed upregulation of a range of genes linked to collagen biosynthesis, including *Adamts2* and the type I collagen-specific integrin complex encoded by *Itga2* (integrin alpha-2 subunit; α2) and *Itgb1* (integrin beta-1 subunit; β1; the full qPCR dataset is available at Gene Expression Omnibus (GEO), under accession number GSE164735), with expression levels consistently higher in male than in female OBVEGFKO cells. ADAMTS2 is highly expressed in a range of tissues rich in type I collagen, including bone, and is involved in the cleavage of the N-terminal sequence from the pro-peptide. Removal of the pro-peptide sequences is essential for the generation of functional collagen monomers that are capable of extracellular fibril assembly, with inherited deficiencies in this protein resulting in skin fragility in patients with dermatosparactic Ehlers-Danlos syndrome (Bekhouche and Colige, 2015; Le Goff et al., 2006). Conversely, the upregulated expression of *ADAMTS2* has been documented in the genomic assessment of fibrous dysplasia (Zhou et al., 2014), which is characterised by overproduction of the ECM specifically in bone. Intriguingly, the skeletal phenotypes observed in the male OcnVEGFKO bones display a number of parallels to those seen in fibrous dysplasia patients, particularly the increased prevalence of woven bone (Burke et al., 2017) containing randomly orientated collagen fibrils, in line with our findings. Complementing this, we also detected a drastic upregulation in the expression of α2β1 integrin mRNA expression in male versus female *Vegf*-deficient cells. Previously, the elevated expression of the integrin α2 subunit has been shown to positively regulate the expression of the *Col1a1* gene, leading to the synthesis of type I collagen (Riikonen et al., 1995). We speculate that the combined effects of upregulated *Itga2* and *Adamts2* expression in the male OBVEGFKO cells may, in fact, be contributing significantly to the excessive and disorganised sex-specific matrix phenotype observed in the OcnVEGFKO bone cortex.

We also noted further sex-specific variations in the mRNA levels of genes associated with angiogenesis. Laminin-1, recognised by α2β1 integrins, exhibits pro-angiogenic effects in the vascular endothelium. Both *Lama1* and *Mmp9* expression exhibited greater upregulation in male than in female OBVEGFKO cells. In particular, elevated levels of MMP9 correlate with osteoporotic bone loss (Haibo et al., 1997), and, given the function of MMP9 in matrix remodelling, these findings indicate possible pathological remodelling effects that could, in part, be responsible for the aberrations in matrix microarchitecture and vascular anomalies in the male OcnVEGFKO bones (Goring et al., 2019). Contrasting with these effects, differential changes in the expression of *Hc* and *Itgae* were detected following OBVEGFKO in male and female OBs. Reductions in HC, in contrast to MMP9, are associated with the pathogenesis of degenerative bone loss (Ehrnthaller et al., 2013). Deficiencies in *Hc* have also been shown to severely reduce the mechanical competence of bone following fracture due to impaired fracture healing (Ehrnthaller et al., 2013). In the current study, we noted sexual dimorphism in the expression of *Hc* following OBVEGFKO *in vitro*, with upregulation of mRNA transcripts detected in female OBs and downregulated expression in male OBs, indicating that additional and alternative mechanisms involving the immune system may also contribute to the alterations to the ECM.

**Fig. 6.**
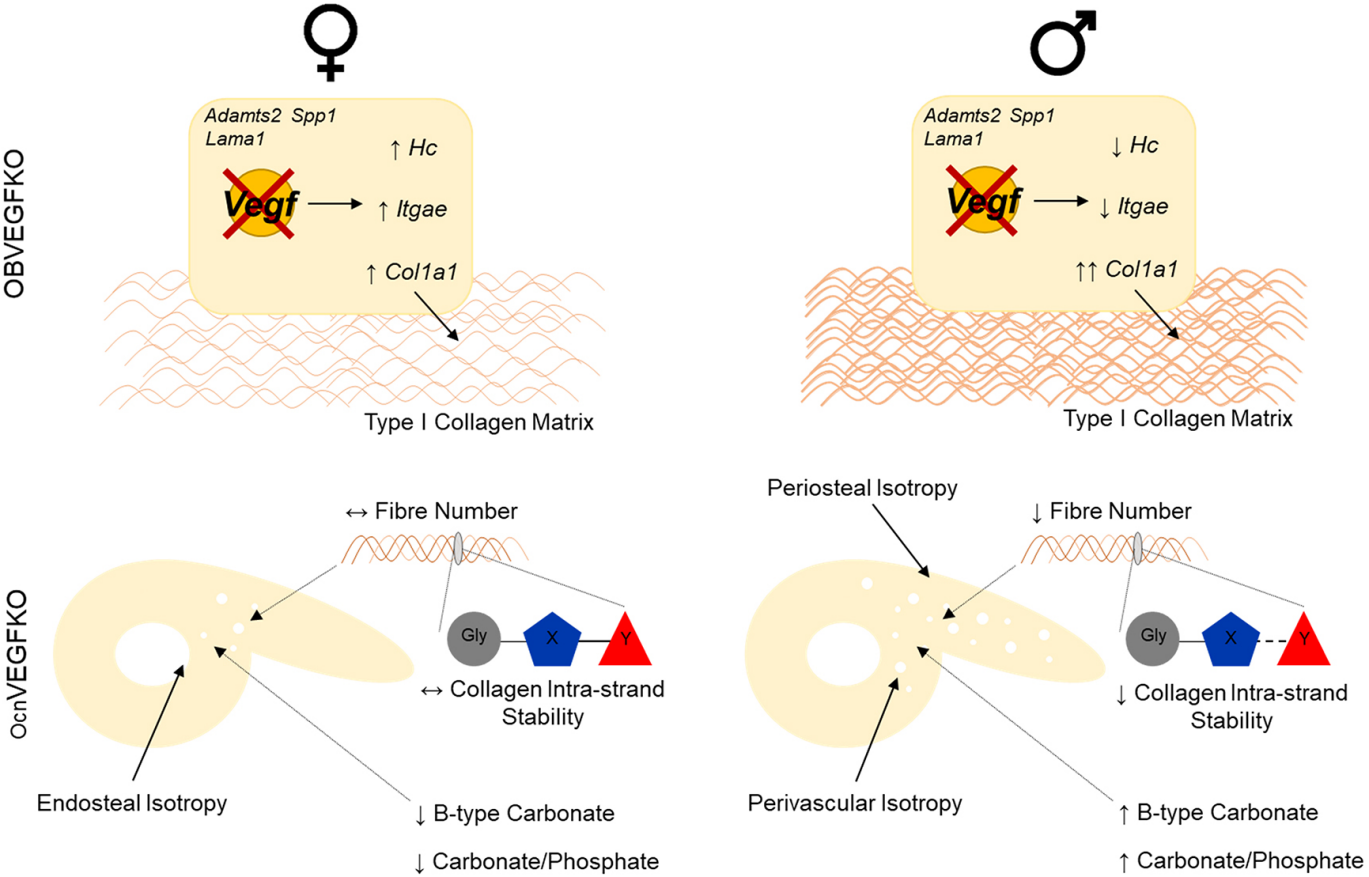
**Multiscale sexual dimorphism of the bone matrix is induced following the deletion of VEGF.** In female OcnVEGFKO bones, localised deformation of collagen fibril arrangement was confined to the endosteal region of the posterior cortex at the TFJ, with no detectable change to collagen fibre number. Collagen intra-strand stability was unaffected; however, reductions in the levels of B-type carbonate were coupled to further sex-specific reductions in the carbonate/phosphate ratio following OcnVEGFKO. In contrast, the loss of VEGF in male OcnVEGFKO bones resulted in modified arrangement of collagen fibrils around the posterior periosteal region and vascular canals, paired with reduction in fibre number and loss of intra-strand stability. These effects were associated with increases in both B-type carbonate and the carbonate/phosphate ratio. These macroscale changes in organisation and nanoscale alterations in composition could be indicative of a direct role of VEGF in the sex-specific regulation of ECM formation and remodelling *in vitro*, identified following the deletion of OB-derived VEGF in male and female cell cultures.

In summary, the present study has demonstrated that the combined application of p-SHG with Raman spectroscopy can provide a quantitative, non-destructive approach to characterise the arrangement and composition of the bone matrix on multiple hierarchical levels (Fig. 6). In the context of VEGF, we have found that its deletion in osteocalcin-expressing cells dramatically affects the bone matrix; hallmarked by pronounced alterations to collagen matrix organisation, localised alterations in fibrillar anisotropy and mineralisation. Alterations in genes linked to ECM also appear to be sex specific in OBs. Enhanced understanding of the sex-specific genetic control of the arrangement of the bone matrix could therefore be used to therapeutically modulate dysfunctional mineralisation at the onset of bone pathology or promote fracture repair processes distinctively in males and females.

## MATERIALS AND METHODS

### Specimen preparation

Tibiae from 16-week-old male and female *Vegf^fl/fl^* (WT) and *Vegf^fl/fl^*; Ocn-Cre (OcnVEGFKO) littermates were derived as previously described (Goring et al., 2019) in accordance with the UK Animals (Scientific Procedures) Act 1986 and regulations set by the UK Home Office. Briefly, tibiae were fixed for 48 h on rotation in 4% formaldehyde, pH 7.4 (from paraformaldehyde; Merck, UK) and dehydrated in ethanol ahead of embedding in poly-methyl methacrylate (PMMA). Then, 5-μm-thick cross sections were cut in the region of the tibial diaphysis termed the TFJ (Fig. 1A,B). This region was selected given the clear and distinctive morphology of the TFJ as a landmark area for uniform interrogation of the ECM and its components (Fig. 1C-E) across the genotypes and sexes (Goring et al., 2019; Javaheri et al., 2015; Núñez et al., 2018). Data presented are representative of sections from three littermate-matched WT and OcnVEGFKO animal pairs.

### SHG microscopy

**Fig. 7.**
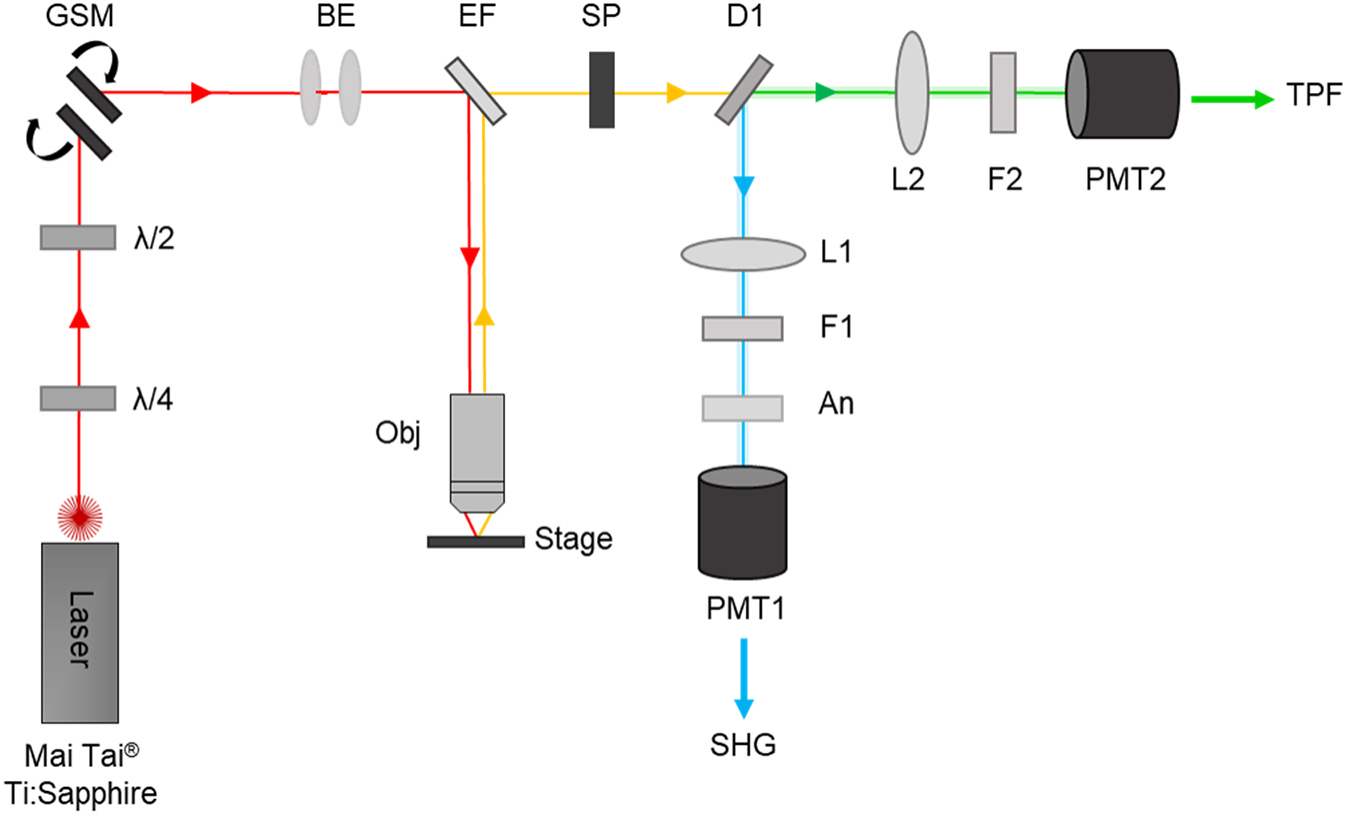
**Schematic of the multiphoton laser scanning system.** The femtosecond pulsed Titanium (Ti):Sapphire laser, tuned to produce an output at 808 nm (red), passes through a quarter (λ/4) and half (λ/2) waveplate allowing control of the polarisation state of the fundamental beam. The beam is directed towards a pair of galvanometric mirrors (GSM) to enable laser scanning, followed by a beam expander (BE) prior to being directed to the stage-mounted sample through an excitation filter (EF) and microscope objective (Obj). The generated sample signal (yellow) is backwards collected through the objective and separated from the excitation wavelength by the same EF and short-pass (SP) filter. Second-harmonic generation (SHG) signal (blue) is isolated from TPEF by a dichroic beam splitter (D1) and directed onto a photomultiplier tube (PMT1) after being focused (L1) and filtered (F1). A linear analyser (An), placed ahead of PMT1 after F1 enables detection of polarisation-dependent SHG emissions. TPEF signals (green) are similarly focused (L2) and filtered (F2) before detection by PMT2.

SHG images were acquired using a home-built multiphoton laser scanning system consisting of a tuneable, pulsed femtosecond laser (Titanium:Sapphire laser; Mai Tai^®^, Spectra-Physics, USA; 710-990 nm, 100 fs, 80 MHz) coupled to an upright Leica DMRB microscope through a pair of galvanometric mirrors for laser scanning (Fig. 7). The fundamental laser beam, tuned at 808 nm for p-SHG microscopy, first passes through a beam expander before being directed towards and focused onto the sample by a microscope objective. The resulting signal is backwards collected by the objective and directed to a dichroic beam splitter (cut-off at 685 nm; FF685-DI02-25X36, Semrock, USA) followed by a short pass-filter (cut-off at 694 nm) allowing for separation of signal from the excitation beam. A second long-pass dichroic beam splitter (cut-off at 458 nm; FF458-DI02, Semrock) in the detection path enables the separation of SHG from TPEF before passing through narrow band-pass filters (400±20 nm for SHG; FB400-40, Thorlabs, USA; and 520±20 nm for TPEF; FBH520-40, Thorlabs) to facilitate the transmission of selected wavelengths prior to their detection by separate photomultiplier tubes (PMTs; H10722-01 and H10722-20, respectively, Hamamatsu, Japan). For circular p-SHG imaging, the polarisation of the fundamental in the excitation path was controlled using a quarter waveplate (λ/4; zero-order, 808 nm, Thorlabs). A half waveplate (λ/2; WPH10M-808, Thorlabs) was further used to correct for ellipticity in the system (generated by non-45° reflections within the scanning system) enabling circular polarisation at the focus. For the determination of anisotropy, p-SHG images were acquired at orthogonal polarisations (parallel and perpendicular to the laser polarisation) using a linear polarisation analyser (LPVISE100-A; 400-700 nm, Thorlabs) which was placed ahead of the PMT in the SHG detection path exclusively. All p-SHG images of bone sections were acquired using either a 20× air objective with a numerical aperture (NA) of 0.7 (Leica, Germany; Figs 2 and 3) or a 63× water immersion objective with an NA of 1.2 (Leica; Fig. S1) in tandem with the MATLAB ScanImage interface (Vidrio Technologies, USA; Pologruto et al., 2003), providing a field of view of ∼313×313 μm or 249×249 μm, respectively. Laser power at the specimen was ∼30 mW. Image acquisition settings included 3× optical zoom with three acquisitions collected over a period of 8-16 ms per line for a 512×512-pixel image. Images were processed using FIJI (Schindelin et al., 2012). Exemplary p-SHG images obtained by circularly and linearly polarised excitation beam are shown in Fig. S2. Owing to the limited lateral field of view, tiled images were taken in 200-μm steps in the *x*- and *y*-planes across the TFJ section, intensity normalised in FIJI then manually restitched using MosaicJ, a semi-automated ImageJ software plugin (Thévenaz and Unser, 2007) ahead of region-specific image analysis.

### Collagen fibre analysis

For the quantitative assessment of collagen fibre number, curvelet transform-fibre extraction (CT-FIRE V2.0; https://loci.wisc.edu/software/ctfire; Bredfeldt et al., 2014)-based analysis was performed on SHG images obtained exclusively using a circularly polarised input. Circular polarisation enables the equal excitation of all fibres independent of their orientation, in a given focal plane. Stitched p-SHG images of entire TFJ sections were segmented manually into the anterior, lateral, medial and posterior quadrants and saved as separate images using FIJI (Fig. 2K) in line with previous work (Núñez et al., 2018). All further regionalised analyses were performed on p-SHG images of the segmented posterior region. Prior to fibre analysis in CT-FIRE, all SHG images were converted to 8-bit format before batch processing using default settings recommended by the developers. Curvelet transform was performed first to de-noise the image and enhance fibre edges followed by the application of the FIRE algorithm to extract the fibre number (Fig. 2K,L). Fibre number was normalised to the area of the segmented posterior quadrant, calculated using the freehand tool and measure function in FIJI.

### Polarisation anisotropy calculation and analysis

For determining anisotropy in collagen fibril orientation, p-SHG images were obtained with the linear polarisation analyser orientated parallel (∥; 0°) and orthogonal (⊥; 90°) relative to the excitation laser polarisation. The SHG anisotropy factor (β), varying from −0.5 to 1 (Chen et al., 2012), was calculated pixel-wise in stitched images of the posterior region of the TFJ using an in-house FIJI script satisfying the following equation: *I*_∥_−*I*_⊥_/*I*_∥_+2*I*_⊥_=β, where *I*_∥_ and *I*_⊥_ indicate the recorded SHG intensity at polarisations parallel and perpendicular, respectively, to the excitation. For endosteal and periosteal anisotropy analysis, measurements were confined to one 120×25 μm-sized ROI box placed on the respective areas per section. Similarly, for perivascular anisotropy analysis, measurements were restricted to five 100×100 μm-sized ROI boxes placed over visible vascular canals.

### BSE-SEM

PMMA-embedded bone blocks were polished ahead of imaging with an EVO MA10 scanning electron microscope (Zeiss, Cambridge, UK) using an acceleration voltage of 20 kV and 49 Pa chamber pressure, as previously described (Goring et al., 2019). Aqueous iodine in ammonium iodide or iodine vapour was used to stain the surface of PMMA blocks to enable the visualisation of cellular detail (Boyde et al., 2014, 2017).

### Raman spectroscopy

Raman spectra were acquired using an InVia^®^ Raman microscope (Renishaw, UK) equipped with a 532 nm continuous-wave solid-state laser with Gaussian beam profile, and a 20× air objective with an NA of 0.4 (Leica), yielding a diffraction limited spot size of ∼815 nm. Incorporated in this system is a Rayleigh edge filter, 2400 lines/mm grating providing spectral resolution of ∼1.06 cm^−1^ and a Renishaw Peltier cooled charged coupled device detector. Intensity and wavenumber calibration of the instrument was achieved as previously described prior to spectral acquisition (Sharma et al., 2020; Smith et al., 2017). Spectra were collected within the ‘Fingerprint region’ from 700 cm^−1^ to 1750 cm^−1^ (Vandenabeele, 2013) using single-point static scans with an exposure time of 10 s, 100% laser power and three accumulations set using WiRE 3.4 (Renishaw). Raman spectra, presented as class means, were obtained from 25 single and random points localised within the posterior cortex, identified as previously described (Núñez et al., 2018) from each bone section. Power at the specimen was ∼37 mW.

### Raman spectral processing and analysis

Cosmic ray artefacts acquired upon acquisition were removed as previously described (Sharma et al., 2020; Smith et al., 2017). IRootLab (Trevisan et al., 2013) was utilised for spectral pre-processing, ahead of spectral deconvolution analysis, wherein class mean Raman spectra were de-noised using Haar wavelets (6-point smoothing) and background corrected by the fitting of 7th order polynomial. Class mean spectra were intensity normalised to the Raman peak at 1450 cm^−1^ corresponding to the CH_2_ wag vibration (Fig. 4A; Fig. S3A). Peak assignment of PMMA contributions in bone Raman spectra were identified following the inspection of unprocessed spectra obtained from visible areas of PMMA, devoid of bone, using the same experimental conditions (Fig. S3B). The 812 cm^−1^ peak was identified as a PMMA artefact and was not analysed further. We note that the 1450 cm^−1^ peak contains contributions of PMMA; thus, in order to normalise for the varying amounts of PMMA detected in each successive point spectra, the 1450 cm^−1^ peak was selected for wavenumber and intensity normalisation, enabling consistency in spectral comparisons across the genotypes and sexes. Peak positions of Raman bands of interest corresponding to ECM and mineral components (proline, 853 cm^−1^; hydroxyproline, 876 cm^−1^; HA, 960 cm^−1^; B-type carbonate, 1070 cm^−1^; Fig. 4A; Fig. S3C) were identified from second-order derivative spectra (Fig. S3D) as previously described (Sharma et al., 2020). Peak areas from class means spectra were extracted by spectral deconvolution, whereby specified bands of interest were fitted with mixed Lorentzian and Gaussian curves using WiRE 4.1 (Renishaw) as previously described (Sharma et al., 2020).

### Isolation and culture of OBs

Primary long-bone OBs from 4-day-old male and female *Vegf^fl/fl^* mice were isolated using the collagenase-collagenase-EDTA-collagenase extraction method as previously described (Orriss et al., 2014). Prior to plating, cells were cultured in complete Phenol Red-free α-Minimum Essential Medium (41061, Gibco, UK) containing 10% heat inactivated fetal bovine serum (FBS; Gibco), 50 μg/ml gentamicin (Merck, UK) and 100 U/ml penicillin and 100 μg/ml streptomycin (Merck) until 80% confluent in 75 cm^2^ flasks (Thermo Fisher Scientific, UK). All cell cultures were maintained in a humidified incubator at 37°C and 5% CO_2_ and used at passage 1.

### Adenoviral transduction

*Vegf* expression was deleted in OBs, plated at a density of 250,000 cells per well, using adenovirus-Cre-GFP (denoted as OBVEGFKO; 1045, Vector Biolabs, USA) at a multiplicity of infection of 100 for 6 days. Viral transduction was performed in complete, Phenol Red-free α-Minimum Essential Medium as previously described (Goring et al., 2019). Adenovirus GFP (denoted as WT; 1060, Vector Biolabs) was used as a control. Cells were stepped down in low serum medium, containing 1% FBS for 24 h, ahead of conditioned medium collection for knockdown validation and RNA lysate collection for gene expression analysis. Fluorescence images of adenoviral transduced cells were acquired using a Deltavision Elite microscope with a 10× air objective and an NA of 0.4, in combination with the SoftWoRx acquisition software (GE Healthcare Sciences, USA).

### Validation of VEGF knockdown

A VEGF sandwich mouse ELISA kit and reagents (ab209882, Abcam, UK) was used to confirm VEGF knockdown in the collected conditioned media from male and female OBVEGFKO cells versus WT cells according to the manufacturer's instructions. Purified total RNA concentration was used to normalise VEGF concentrations quantified by the ELISA.

### qPCR array

RNA was isolated and purified using the Monarch^®^ Total RNA Miniprep kit (New England Biolabs, USA), according to the manufacturer's instructions, and 500 ng RNA per preparation was reverse transcribed into cDNA using a GoScript Reverse Transcriptase kit (Promega, USA). Gene expression changes associated with ECM and adhesion molecules in male and female WT and OBVEGFKO samples were screened using an RT^2^ Profiler™ PCR Array (PAMM-013Z, 330231, Qiagen, Germany) and the StepOnePlus Real-Time PCR system (Applied Biosystems, UK) following the manufacturers’ instructions. Ct values were normalised to housekeeping genes included in the array, and relative expression was calculated using the ΔΔCt method (Livak and Schmittgen, 2001).

### Statistical analysis

All investigators were blinded to the genotype and sex of animals throughout experimentation until data analysis. Data are expressed as the mean±s.e.m. Genotype effects between WT and OcnVEGFKO groups and sex effects between male and female groups were assessed using paired Student's *t*-test. Interaction between sex and genotype was determined using two-way ANOVA with Tukey's post hoc test conducted for further comparisons. All statistical analysis was performed using Prism 8 (GraphPad Software, California, USA). *P*<0.05 was considered statistically significant.

## Supplementary Material

Supplementary information
