# Supplementary material for: Multiscale molecular profiling of pathological bone resolves sexually dimorphic control of extracellular matrix composition
Source: Dis Model Mech. 2021 Mar 17;14(3):dmm048116. doi: 10.1242/dmm.048116 (PMC7988766; doi:10.1242/dmm.048116)
Supplement: Supplementary information [file dmm-14-048116-s1.pdf]

Supplementary Information

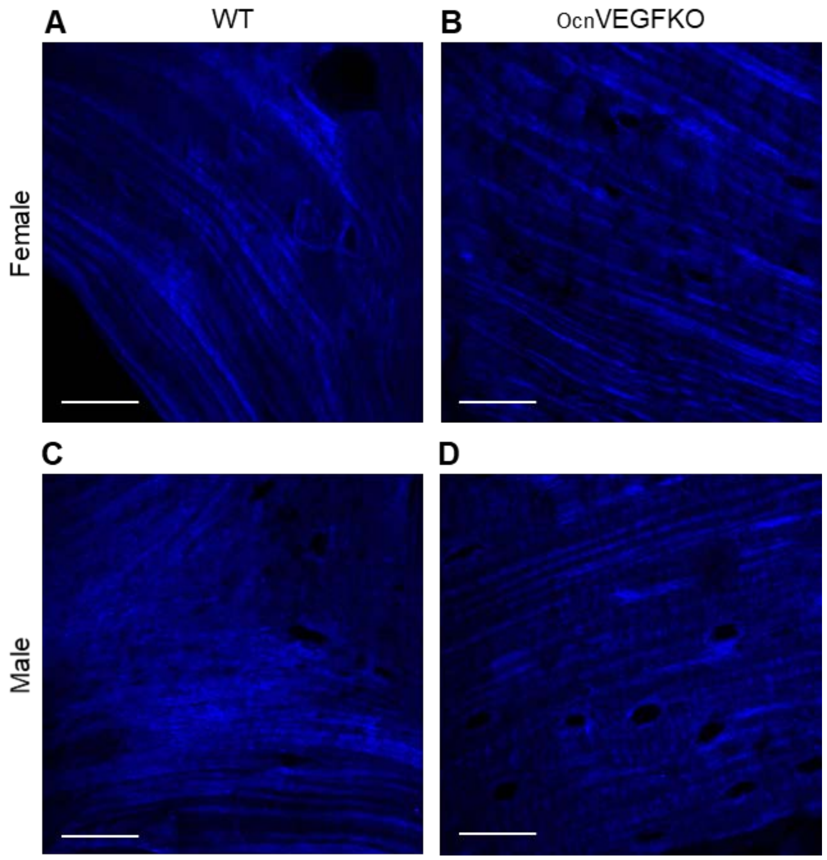

Fig. S1. High magnification p-SHG images of PMMA embedded sections reveal collagen fibres in female WT (A), female OcnVEGFKO (B), male WT (C) and male OcnVEGFKO (D) sections. Scale bars represent 50 µm.

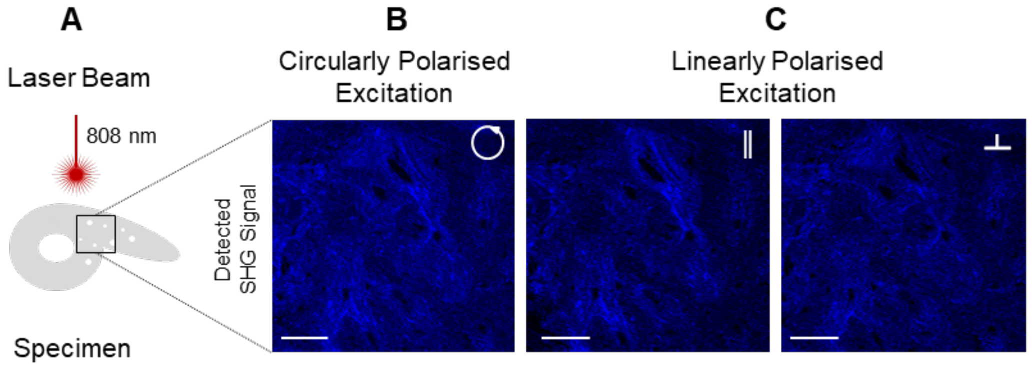

Fig. S2. Visualisation of bone matrix collagen fibres is polarisation dependent. SHG images of PMMA embedded sections were acquired by tuning the wavelength of the fundamental excitation laser to 808 nm (A). Circular polarisation of the fundamental beam, achieved by modulation of λ/4 and λ/2 waveplates, enabled uniform excitation of collagen fibrils (B) orientated in all directions across the specimen. Linear polarisation enables excitation of collagen fibrils that are orientated parallel (C; ‖, left) or orthogonal (C; ⊥, right) to polarisation of the fundamental laser, showing maximum intensity with alignment at 0 ° (‖) and minimum at 90 ° (⊥). Scale bars represent 50 μm.

Disease Models & Mechanisms • Supplementary information

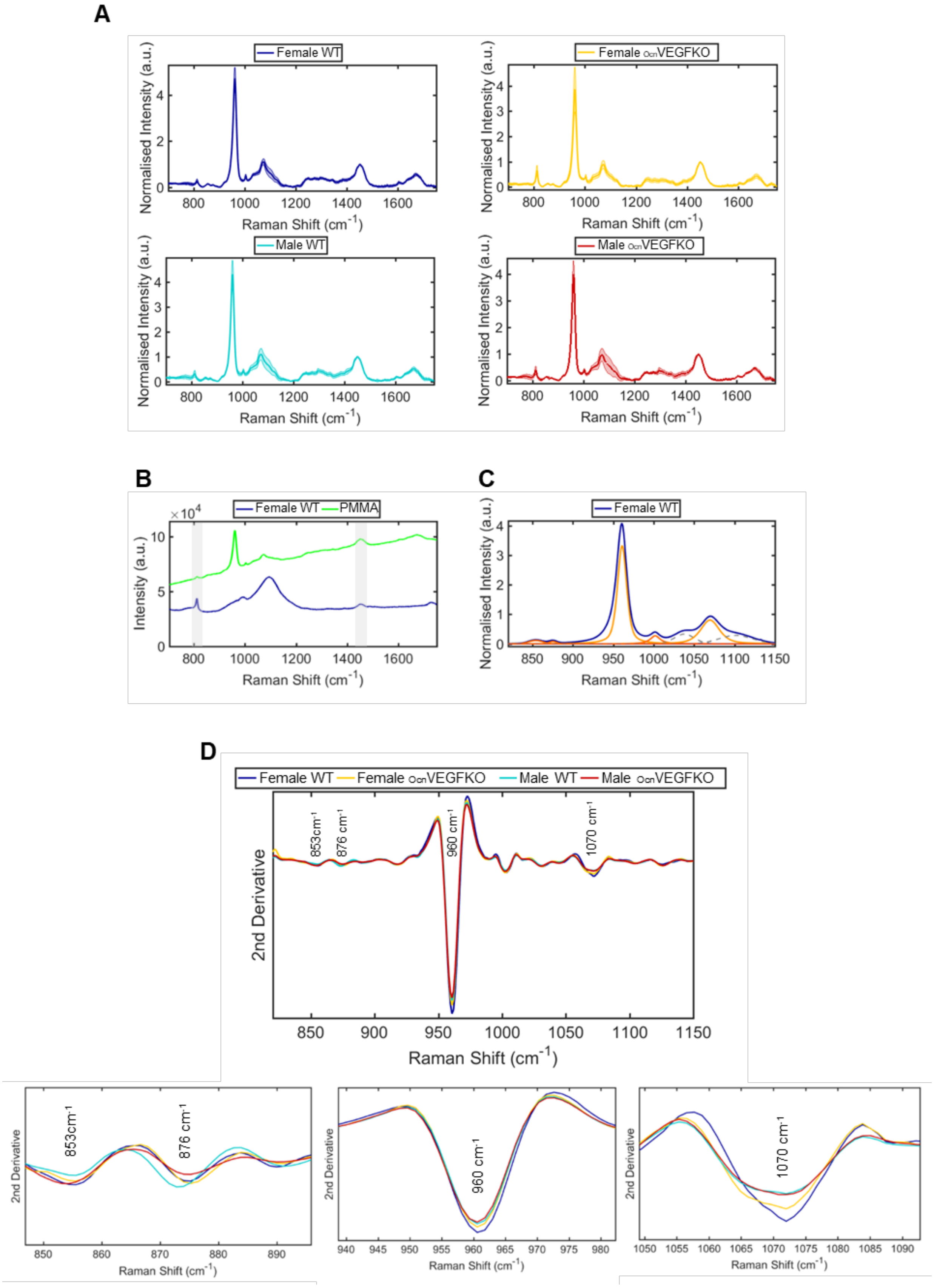

Fig. S3. Raman spectra collected within the fingerprint region from 700 cm^{-1} to 1750 cm^{-1} from the posterior region of three littermate matched female WT, female OcnVEGFKO, male WT and male OcnVEGFKO TFJ sections are presented as class means of n=75 single spectra with SD displayed (A).

Disease Models & Mechanisms • Supplementary information

PMMA contributions in bone Raman spectra are highlighted in raw, unprocessed spectra ahead of deconvolution analysis. Peaks of interest associated with the ECM (proline, 853 cm^{-1} and hydroxyproline, 876 cm^{-1} in type I collagen) and mineral (HA, 960 cm^{-1} and B-type carbonate, 1070 cm^{-1}), displayed as orange lines (C) are identifiable in bone Raman spectra following the calculation of second derivative spectra (D). Zoomed spectral peaks of interest identified in the second derivative spectra shown underneath (D) were analysed further by spectral deconvolution.

Disease Models & Mechanisms • Supplementary information
